# Antibiotic drug usage in pigs in Germany—Are the class profiles changing?

**DOI:** 10.1371/journal.pone.0182661

**Published:** 2017-08-25

**Authors:** Franziska Schaekel, Thomas May, Julia Seiler, Maria Hartmann, Lothar Kreienbrock

**Affiliations:** 1 Department of Biometry, Epidemiology and Information Processing, WHO Collaborating Centre for Research and Training for Health at the Human-Animal-Environment Interface, University of Veterinary Medicine, Hannover, Germany; 2 QS, Qualität und Sicherheit GmbH, Bonn, Germany; Animal and Plant Health Agency, UNITED KINGDOM

## Abstract

The development of antimicrobial resistance is triggered by the use of antibiotic drugs. Therefore, the consumption of antibiotics in livestock is monitored, and different measures may be applied if the usage of antibiotic drugs seems inappropriate. Unfortunately, the surveillance of antibiotic consumption is not standardised, and surveillance systems differ. In Germany, the food quality assurance system QS Qualität und Sicherheit GmbH (QS) began the documentation of antibiotic drug usage in pigs in 2012 in a private economic based database, and for its members, documentation has been mandatory in all pig age groups since 2014. In this investigation, we calculated the distribution of the antibiotics use per pig age group and half-year, and the percentage of the active substances used from overall treatments within German pig holdings from 1 July, 2013 to 30 June, 2015. In fattening pigs, the median of the treatment frequency is 4.3 in 2013–2 and exhibits a decreasing trend in this time period up to 2.1 in 2015–1. In weaners the median ranged between 11.3 in 2014–2 and 5.8 in 2013–2. The median of sucklers varies between 21.6 and 25.0. In sucklers and weaners, a clear temporal trend is not seen to date. The share of the active substances differs between the age groups. In fattening pigs, mostly tetracyclines and penicillines were used, occurring in approximately 60% of the total treatments. In weaners, amoxicillin and colistin have the highest shares of the treatment frequency, at approximately 60%. The treatment frequencies of macrolides and penicillines have the highest share in sucklers.

## Introduction

The relationship between the development of resistance in bacteria and the use of antibiotics is well-known [1]. Resistant bacteria are a problem for both human and veterinary medicine. In Germany, being aware of the risk of resistance developing from the use of antibiotics, the Federal Veterinary Chamber ("Bundestierärztekammer"), in cooperation with the working group of the Chief Veterinary Officers of the German federal states ("Arbeitsgemeinschaft der Leitenden Veterinärbeamten"), has published guidelines for the prudent use of antibiotics in animals since 2000. This document describes the prudent use and antibiotic stewardship for veterinarians [2].

Another starting point to fight antimicrobial resistance is to reduce the use of antibiotics in veterinary medicine in general. Therefore monitoring the use of antibiotics is essential. For that purpose, different systems were established in the recent years for example in Austria [3], Denmark [4], the Netherlands [5, 6], Norway [7] and Sweden [8]. A centre of expertise was founded in Belgium in recent years whose goals are not only monitoring of antibiotic use in animals and benchmarking but also the reduction of antibiotic use and promotion of alternatives. Moreover a surveillance of resistance to antibiotics should be implemented [9] ESVAC has also published guidance for data collection by species [10].

In Germany as well systems for data collection and reduction of antibiotics are established for farm animals in the recent years. The amount of active substances from sales data and the frequency of use of antibiotics is monitored via different systems. Sales data is available by a regulatory act [11] since its first documentation from 2011 onwards. As a scientific project, the "Veterinary consumption of antibiotics" (VetCAb)- Monitoring has offered data from the whole of Germany since 2007 for farm animals [12, 13]. Since 2012, QS (QS Qualität und Sicherheit GmbH) has been running an antibiotic monitoring system for its members for all pig age groups, beef cattle and poultry. QS is a private company that has organised a quality assurance system that covers all trade levels of meat and meat products from farmers to retail since 2001 [14]. Obligatory monitoring was adopted by the 16^th^ amendment of the German Pharmaceuticals Act ("Arzneimittelgesetz", AMG) in 2014 which regulates the official monitoring of the use of antibiotic drugs for weaners and fattening pigs, beef cattle and poultry. Also it formed the basis for the implementation of benchmarking of farms. But, no scientific evaluation of the data is permitted by law. Therefore, for now the scientific project VetCAb and the private company QS data on use of antibiotic can be used for different evaluations only.

This paper focuses on the antibiotic monitoring system of the QS and takes the different age groups of pigs into consideration. The aim of the study is to describe the use of the various antibiotic substances in the entire German pork production and examine its temporal development.

## Materials and methods

### QS monitoring system for antibiotic use

Approximately 32,913 national Farm-IDs from pig farmers in Germany are connected to the QS system; as such, approximately 95% of the pigs slaughtered in Germany are related to the QS system. The collection of data regarding the use of antibiotics in fattening pigs began in 2012. Since 2014, the input of data has also been mandatory for farms holding weaners and sucklers [15].

As proposed by Jensen et al. [16] and other authors we analysed the different age groups independently. By QS definition, a suckler is a pig that is suckled, a weaner is a pig post-weaning that weighs less than 30 kg, and a fattening pig is a pig with a body weight between 30 and 120 kg [17]. These weight groups for pigs are similar to the age categories defined by ESVAC [18].

The prescription of antibiotics for livestock in Germany is subject to various legal regulations. For example, only veterinarians are allowed to prescribe antibiotics after examination of affected animals [19]. The instruction and information pertaining to a treatment has to be noted down on special forms ("antibiotics application and delivery form", ADF) in duplicate, one of which is given to the farmer, while the second form remains with the veterinarian. The ADF contains information about the number and type of animals being treated, the name of the drug, the number of days treated, the drug dosage, the type of application and various additional information. With this method, the daily dose used (per drug and per class) is documented directly. The veterinarian classifies the treated pigs to the appropriate age class. In addition in the QS system the veterinarian is responsible for the correct and complete input of the ADFs in the database. If no antibiotic treatment has taken place in one half year per farm and age group, a "no use" information has to be entered in the database as well.

### Preparation of data set

Different plausibility checks were performed at the input process in the database, for example the "no antibiotic use" input is only possible if no ADF is entered in the database. Furthermore employees of QS give feedback to the responsible veterinarian about potentially implausible or missing values which should be corrected afterwards by the veterinarian.

All data was pseudonymised by QS by using codes instead of full names and addresses to ensure data privacy. After receiving the data set from QS the variables needed for calculation were checked once more and ADFs with "number of days treated" = 0, "number of animals treated" = 0, "amount of substance" = 0 or "population size" = 0 were excluded from the evaluation which effects about 3,000 ADFs.

A list of long acting products was made available from QS. For those products the number of days treated is extended by the veterinarian's individual definition. For reporting of results on active substances this correction factors were taken into account.

### Statistical evaluation of the data

Antibiotic usage was calculated by means of the treatment frequency TF per half year, which relates the number of used daily doses to the farm size, i.e.

$$\text{TF}=\frac{\text{nUDD}}{\text{farm size }}.$$

[12, 13]. The information needed to calculate the number of used daily doses (nUDD = number of days treated × number of active substances applied × number of animals treated) is included in the ADFs directly, so the number of used daily dose does not need to be estimated with an average animal weight or other surrogates. The population size is defined as the average number of housed animals per age class documented by the farmer in the entire QS system. To evaluate the treatment frequency in sucklers, the population size is linked to the average number of sows housed.

The definition of TF follows the concept of the (cumulative) incidence in epidemiology by relating events (here nUDD) to a (fixed) population size (here the farm size). As nUDD may be re-arranged

$$\begin{array}{ll} \text{nUDD} & =\frac{\text{amount used}}{\text{animal weight}\times \text{UDD }}=\frac{\text{amount used}}{\text{animal weight}\times \frac{\text{amount used}}{\text{animals treated}\times \text{animal weight}\times \text{days treated}}\;}\\ & =\text{ animals treated}\times \text{days treated} \end{array}$$

TF describes the number of days all animals within the stock are treated in average. This is the same as Timmermanns et al. [20], Persoons et al. [21] and others are calculating by introducing average body weights to the stock treated.

This is in a slight contrast to other definitions, which follow the concept of an incidence density, where nUDD is divided by a farm-individual time-at-risk.

Pharmaceuticals or treatments containing two or more different active substances are entered into the calculation with a value of two, or more. The combination of sulfonamides with trimethoprim, ampicillin and cloxacillin, benzylpenicillin-benzathin and benzylpenicillin-procain, as well as benzylpenicillin-kalium and benzylpenicillin-procain are interpreted as one active substance.

The treatment frequency is calculated for every age group per national Farm-ID and half year as its whole as well as for every active substance separately. To illustrate the distribution of treatment frequencies, an empirical density function was approximated (restricted to treatment frequencies smaller than or equal to 100) by means of a negative binomial model.

The percentage of the treatment frequency of an active substance per total treatment is calculated using a unilateral alpha trimmed data set (1%) for a more robust statistical inference [22]. To this end, the total treatment frequency and the treatment frequency per active substance are summed up. From the total treatment frequency, the percentage of each active substance is calculated [23]. This calculation is performed separately for each age class and half year.

All ADF information and basic farm data were linked by national Farm-ID and evaluated with SAS^®^, version 9.3 TS level 1M2 (SAS Institute Inc., Cary, NC, United States).

## Results

### Description of the study population

In total, 924,771 ADFs (100%) were made available from QS during the study period. The number of participating holdings in each age group increased steadily until 2014–2 (Table 1). Consequently, the number of ADFs per age group also rose. In 2015–1, a slight decrease in the number of participating holdings was seen in weaners and fattening pigs. After assignment to basic farm data, and readability and plausibility checks, 891,925 ADFs (96.45%) could be included in the evaluation. It is important to note that the number of holdings, especially in sucklers and weaners, in 2013–2 was very small in comparison to the other half-years. From 2013–2 till 2014–2, the number of holdings with no treatment per half-year increased, but then decreased in 2015–1. This trend was observed in all three age groups.

**Table 1:** Age groups and number of ADFs included in the study.

age groups	half-year	number of holdings	number of ADF′s	average number of ADF′s per age group	number of holdings with no recorded treatment	% of holdings with no recorded treatment
**sucklers**	2013–2	374	3,867	10.3	40	1.5
	2014–1	4,815	40,795	8.5	388	8.1
	2014–2	6,727	71,803	10.7	443	6.6
	2015–1	6,812	77,793	11.4	319	4.7
**weaners**	2013–2	522	3,395	6.5	51	9.8
	2014–1	6,048	57,805	9.6	750	12.4
	2014–2	8,577	98,132	11.4	1,159	13.5
	2015–1	8,293	84,914	10.2	759	9.2
**fattening pigs**	2013–2	9,588	70,926	7.4	791	8.3
	2014–1	16,960	116,798	6.9	2,958	17.4
	2014–2	20,374	140,619	6.9	3,645	17.9
	2015–1	19,324	125,078	6.5	2,770	14.3

### General trends for treatment frequency

**Fig 1:**
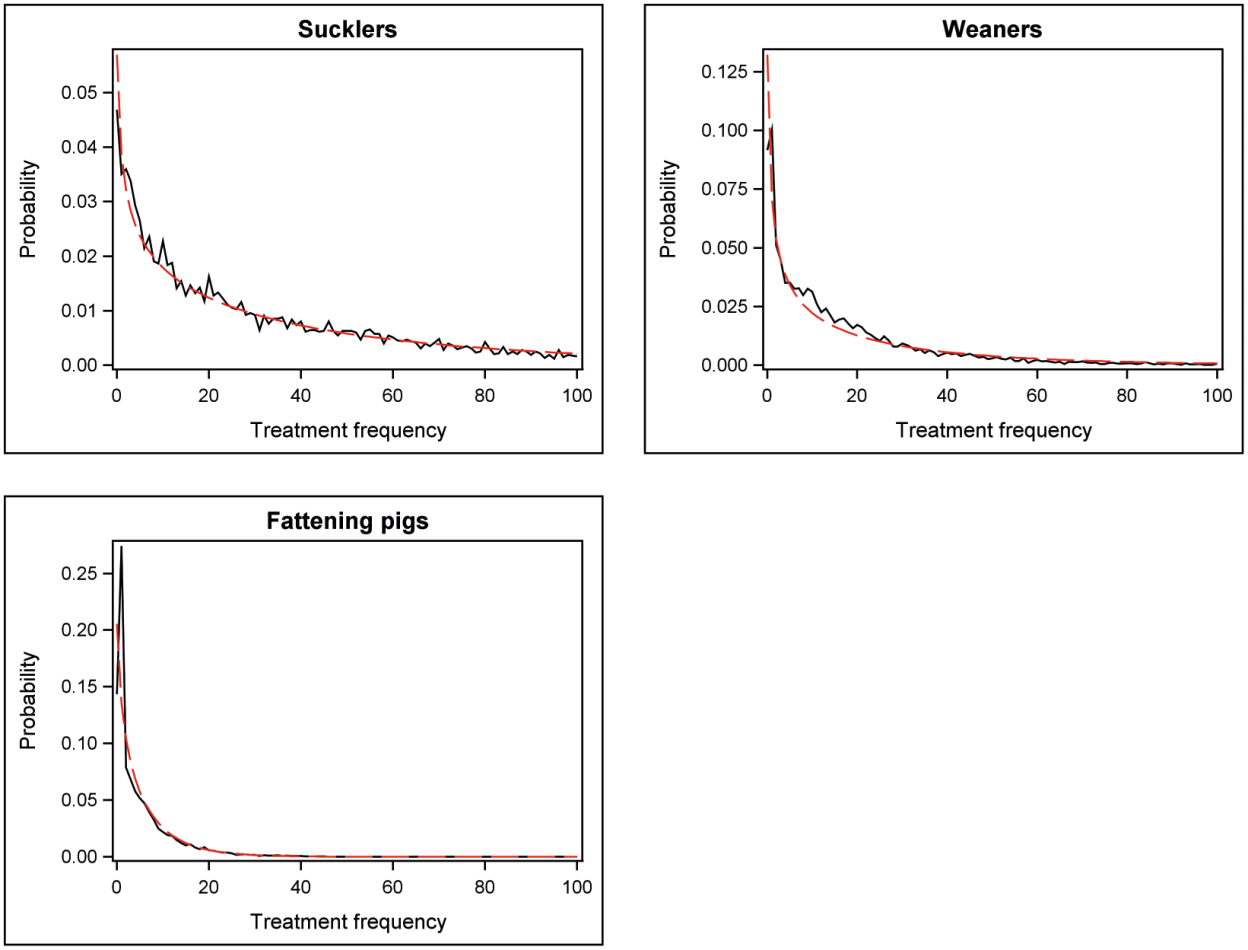
Negative binomial adjusted distribution of the relative treatment frequency for sucklers, weaners and fattening pigs in 2015–1 ---- model approximation, ____ empirical distribution.

The distribution of the relative treatment frequency in all three age groups is shown for 2015–1 as an example in Fig 1. Treatment frequencies, in general, follow a negative binomial distribution; however in fattening pigs, this fit is less optimal due to some zero-inflation in the empirical distribution.

Statistical measures of the treatment frequency distribution for the observation period are shown in Table 2. Extended maxima might indicate an input error in the entire database, and since we cannot exclude this possibility, these values were not interpreted (alpha-trimming). Furthermore, the quartiles of the treatment frequency were not influenced by these values. The zeros in the minimum and in the 5% quartile resulted from holdings where no antibiotic use was recorded. Moreover, it can be observed that the values in sucklers were noticeably higher than in weaners and fattening pigs. This effect is due to the scaling of the reference population for sucklers as the average number of housed sows.

**Table 2:** Statistical measures of the treatment frequency per age group and half-year.

		treatment frequency						
age group	half year	number of holdings	minimum	5%-percentile	median	upper quartile	95%-percentile	maximum
**sucklers**	**2013–2**	374	0	0	21.6	60.8	170.7	664.3
	**2014–1**	4,815	0	0	18.3	45.3	122.7	1,249.0
	**2014–2**	6,727	0	0	25.0	57.2	133.0	3,394.0
	**2015–1**	6,812	0	0.2	23.0	55.7	150.8	1,322.0
**weaners**	**2013–2**	522	0	0	5.8	14.3	55.7	196.6
	**2014–1**	6,048	0	0	9.7	26.2	74.6	3,076.0
	**2014–2**	8,577	0	0	11.3	29.7	76.9	6,118.0
	**2015–1**	8,293	0	0	9.4	22.1	56.8	29,550.0
**fattening pigs**	**2013–2**	9,588	0	0	4.3	11.6	30.4	7,700.0
	**2014–1**	16,960	0	0	3.4	10.6	29.4	27,801.0
	**2014–2**	20,374	0	0	3.0	9.6	26.1	490.0
	**2015–1**	19,324	0	0	2.1	6.7	19.0	425.0

We found a decrease in the median from 2013–2 (4.3) to 2015–1 (2.1) in fattening pigs. In weaners, we found an increase of the median up to 11.3 in 2014–2, and then a decline in 2015–1 to 9.4. The median of the sucklers showed an alternating trend, with a higher median in 2015–1 compared to 2013–2 and 2014–1. Similar trends were found in the 75% and 95% quartile of all age groups.

### Trends in treatment frequency by antibiotic drug class

Treatment frequencies and related statistical measures were stratified into twelve antibiotic drug classes. Due to the age group and indication, the distributional patterns differed substantially. Drug classes which were used in many holdings, like penicillines, had a relatively similar distribution of treatment frequency compared to the overall treatment frequency. The 2014–1 median of penicillines was 3.5 in sucklers, 0.6 in weaners and 0.1 in fattening pigs (not shown in detail). In drug classes which were used in fewer holdings, the median was zero. In polypeptides, for example, the upper quartile in sucklers and fattening pigs was zero as well; there are only figures above zero in the 95% quartile. Because all drug classes (except penicillines) were used in less than 50% of the holdings, the percentage of the treatment frequency for all drug classes and active substances per total treatments was analysed in depth. The three age groups were considered separately (Tables 3–5).

**Table 3:** Percentage of the treatment frequency per active substance of the total treatments in sucklers (%).

Drug class Active substance	2013–2	2014–1	2014–2	2015–1
**Aminoglykosides**	**10.05**	**9.45**	**8.09**	**8.92**
Apramycin	1.09	0.87	0.58	0.61
Dihydrostreptomycin	7.92	6.05	5.39	6.43
Gentamicin	0.45	1.20	1.25	1.29
Kanamycin	0	0.00	0-	0
Neomycin	0.39	0.57	0.28	0.09
Paromomycin	0	0	0	0.00
Spectinomycin	0.21	0.76	0.59	0.51
**3**^**rd**^ **and 4**^**th**^ generation Cephalosporins*	**3.82**	**14.34**	**15.31**	**16.52**
Cefquinom	0.28	1.03	0.87	0.90
Ceftiofur	3.54	13.31	14.44	15.62
**Fenicoles**	**0.02**	**0.33**	**0.33**	**0.38**
Florfenicol	0.02	0.33	0.33	0.38
**Fluoroquinolones**	**2.06**	**6.71**	**6.66**	**7.08**
Danofloxacin	0.10	0.52	0.53	0.60
Difloxacin	0	0	0.00	0
Enrofloxacin	1.77	5.73	5.73	5.96
Marbofloxacin	0.19	0.47	0.40	0.51
**Lincosamides**	**0.14**	**0.66**	**0.45**	**0.34**
Lincomycin	0.14	0.66	0.45	0.34
**Makrolides**	**9.96**	**15.92**	**17.62**	**27.29**
Erythromycin	0-	0.00	0.01	0.00
Tildipirosin	0.30	0.86	0.72	0.82
Tilmicosin	0-	0.07	0.08	0.04
Tulathromycin	8.44	13.81	16.19	25.98
Tylosin	1.21	1.18	0.62	0.45
Tylvalosin	0	0-	0.00	0-
**Penicillines**	**43.95**	**39.41**	**42.68**	**34.28**
Amoxicillin	35.17	31.16	35.17	26.55
Ampicillin	0	0.01	0.00	0.00
Benzylpenicilin	8.78	8.25	7.48	7.72
Cloxacillin	0	0	0.00	0
Phenoxymethylpenicilin	0	0.00	0.01	0.00
**Pleuromutilins**	**0.03**	**0.10**	**0.15**	**0.08**
Tiamulin	0.03	0.10	0.15	0.08
**Polypeptides**	**12.53**	**5.78**	**3.64**	**2.15**
Colistin	12.53	5.78	3.64	2.15
**Potentiated sulfonamides**	**3.87**	**1.20**	**0.53**	**0.35**
Sulfadiazin and Trimethoprim	0.54	0.10	0.02	0.04
Sulfadimethoxin and Trimethoprim	0	0.12	0.04	0.01
Sulfadimidin and Trimethoprim	0.03	0.12	0.14	0.10
Sulfadoxin and Trimethoprim	0.07	0.15	0.12	0.15
Sulfamethoxazol and Trimethoprim	3.23	0.71	0.21	0.06
**Sulfonamides**	**0**	**0.00**	**0.00**	**0.00**
Sulfadimidin	0	0.00	0	0.00
Sulfadoxin	0	0.00	0.00	0
Sulfamethoxpyridazin	0	0	0	0.00
**Tetracyclines**	**13.56**	**6.09**	**4.55**	**2.60**
Chlortetracyclin	1.37	1.55	1.45	0.68
Doxycyclin	8.84	1.48	0.71	0.30
Oxytetracyclin	1.06	2.08	1.86	1.49
Tetracyclin	2.30	0.98	0.53	0.13
**n holdings**	**370**	**4,767**	**6,659**	**6,743**
**sum treatment frequencies in %**	**100**	**100**	**100**	**100**
**sum treatment frequencies**	**16,034.53**	**151,139.88**	**252,850.49**	**264,446.16**

* Cephalosporines of the 1st and 2nd generation as well as Cefoperazon, valnemulin, sulfaclozin, sulfadimethoxin, sulfaquinoxalin and sulfathiazol were not used in sucklers in this study.

**Table 4:** Percentage of the treatment frequency per active substance of the total treatments in weaners (%).

Drug class Active substance	2013–2	2014–1	2014–2	2015–1
**Aminoglykosides**	**2.53**	**2.99**	**2.87**	**2.82**
Apramycin	0.34	0.21	0.24	0.28
Dihydrostreptomycin	0.35	0.30	0.21	0.10
Gentamicin	0.10	0.11	0.08	0.10
Kanamycin	0	0.00	0.00	0
Neomycin	0.87	1.51	1.54	1.55
Spectinomycin	0.87	0.87	0.81	0.78
**3**^**rd**^ **and 4**^**th**^ generation Cephalosporins*	**0.71**	**0.72**	**0.60**	**0.56**
Cefoperazon	0	0-	0	0.00
Cefquinom	0.47	0.18	0.13	0.15
Ceftiofur	0.24	0.54	0.46	0.42
**Fenicoles**	0.28	0.28	0.26	0.41
Florfenicol	0.28	0.28	0.26	0.41
**Fluoroquinolones**	**1.47**	**1.65**	**1.53**	**1.50**
Danofloxacin	0.02	0.18	0.23	0.12
Enrofloxacin	1.20	1.22	1.08	1.17
Marbofloxacin	0.25	0.25	0.22	0.21
**Lincosamides**	**1.15**	**1.22**	**1.31**	**1.29**
Lincomycin	1.15	1.22	1.31	1.29
**Makrolides**	**9.27**	**9.70**	**8.40**	**8.11**
Erythromycin	0	0.00	0.00	0.01
Tildipirosin	0.06	0.21	0.19	0.20
Tilmicosin	0.31	1.24	1.22	1.23
Tulathromycin	1.63	1.54	1.49	2.21
Tylosin	7.23	6.58	5.37	4.42
Tylvalosin	0.03	0.12	0.13	0.05
**Penicillines**	**33.05**	**29.76**	**32.21**	**31.78**
Amoxicillin	32.28	28.93	31.58	31.38
Ampicillin	0.06	0.15	0.14	0.12
Benzylpenicilin	0.71	0.67	0.49	0.28
Cloxacillin	0	0	0.00	0.00
**Pleuromutilins**	**0.19**	**1.16**	**1.07**	**1.30**
Tiamulin	0.19	1.16	1.07	1.30
**Polypeptides**	**25.56**	**30.16**	**29.50**	**30.80**
Colistin	25.56	30.16	29.50	30.80
**Potentiated sulfonamides**	**4.34**	**4.44**	**4.06**	**3.57**
Sulfadiazin and Trimethoprim	0.67	0.10	0.08	0.08
Sulfadimethoxin and Trimethoprim	0	0.18	0.10	0.13
Sulfadimidin and Trimethoprim	0.01	0.05	0.03	0.03
Sulfadoxin and Trimethoprim	0.05	0.08	0.07	0.09
Sulfamethoxazol and Trimethoprim	3.61	4.02	3.78	3.24
**Sulfonamides**	**0.12**	**0.04**	**0.04**	**0.07**
Sulfadimidin	0.12	0.04	0.04	0.07
Sulfamethoxpyridazin	0	0	0.00	0.00
Sulfathiazol	0	0	0	0.00
**Tetracyclines**	**21.32**	**17.87**	**18.15**	**17.79**
Chlortetracyclin	5.61	3.30	3.43	3.01
Doxycyclin	12.87	9.23	9.94	10.29
Oxytetracyclin	0.13	0.35	0.28	0.27
Tetracyclin	2.72	4.99	4.49	4.21
**n holdings**	**517**	**5,987**	**8,489**	**8,212**
**sum treatment frequencies in %**	**100**	**100**	**100**	**100**
**sum treatment frequencies**	**6,466.40**	**108,614.87**	**169,616.56**	**125,623.31**

*Cephalosporines of the 1st and 2nd generation as well as Paromomycin, phenoxymethylpen, difloxacin, valnemulin, sulfaclozin, sulfadimethoxin, sulfadoxin and sulfaquinoxalin were not used in weaners in this study.

**Table 5:** Percentage of the treatment frequency per active substance of the total treatments in fattening pigs (%).

Drug class Active substance	2013–2	2014–1	2014–2	2015–1
**Aminoglykosides**	**1.89**	**2.34**	**2.65**	**2.48**
Apramycin	0.01	0.03	0.01	0.02
Dihydrostreptomycin	0.12	0.08	0.04	0.02
Gentamicin	0.01	0.03	0.04	0.03
Kanamycin	0	0.00	0.00	0.00
Neomycin	0.82	1.10	0.98	1.00
Spectinomycin	0.92	1.12	1.58	1.41
**3**^**rd**^ **and 4**^**th**^ generation Cephalosporines*	**0.28**	**0.27**	**0.21**	**0.29**
Cefoperazon	0	0	0	0.00
Cefquinom	0.17	0.18	0.16	0.21
Ceftiofur	0.12	0.10	0.05	0.08
**Fenicoles**	**0.47**	**0.47**	**0.54**	**0.65**
Florfenicol	0.47	0.47	0.54	0.65
**Fluoroquinolone**	**1.74**	**1.89**	**1.95**	**2.29**
Danofloxacin	0.16	0.16	0.16	0.21
Enrofloxacin	1.15	1.23	1.24	1.54
Marbofloxacin	0.43	0.51	0.55	0.55
**Lincosamides**	**3.90**	**4.29**	**5.08**	**5.30**
Lincomycin	3.90	4.29	5.08	5.30
**Makrolides**	**16.54**	**15.52**	**14.30**	**14.61**
Erythromycin	0.00	0.01	0.01	0.01
Tildipirosin	0.22	0.21	0.19	0.20
Tilmicosin	0.45	0.54	0.47	0.46
Tulathromycin	0.22	0.28	0.23	0.49
Tylosin	15.59	14.40	13.34	13.43
Tylvalosin	0.05	0.09	0.06	0.03
**Penicillines**	**26.39**	**27.17**	**27.26**	**27.57**
Amoxicillin	25.46	26.32	26.44	27.08
Ampicillin	0.12	0.05	0.07	0.06
Benzylpenicilin	0.81	0.79	0.75	0.43
Cloxacillin	0	0	0	0.00
**Pleuromutilins**	**3.12**	**3.38**	**3.95**	**4.68**
Tiamulin	3.12	3.38	3.95	4.68
Valnemulin	0	0.00	0.00	0
**Polypeptides**	**7.29**	**8.54**	**7.83**	**7.63**
Colistin	7.29	8.54	7.83	7.63
**Potentiated sulfonamides**	**7.95**	**6.68**	**5.70**	**4.00**
Sulfadiazin und Trimethoprim	0.02	0.06	0.05	0.04
Sulfadimethoxin und Trimethoprim	0.34	0.20	0.21	0.21
Sulfadimidin und Trimethoprim	0.05	0.06	0.06	0.04
Sulfadoxin und Trimethoprim	0.07	0.04	0.04	0.06
Sulfamethoxazol und Trimethoprim	7.47	6.32	5.34	3.65
**Sulfonamides**	**0.31**	**0.20**	**0.23**	**0.25**
Sulfaclozin	0.01	0	0	0
Sulfadimethoxin	0.00	0	0	0
Sulfadimidin	0.29	0.20	0.22	0.25
Sulfamethoxpyridazin	0.00	0.00	0.00	0.00
Sulfaquinoxalin	0-	0.00	0.00	0.00
Sulfathiazol	0	0.00	0	0
**Tetracyclines**	**30.11**	**29.23**	**30.31**	**30.24**
Chlortetracyclin	5.08	4.59	4.62	4.08
Doxycyclin	15.62	16.95	18.84	20.37
Oxytetracyclin	0.22	0.22	0.20	0.30
Tetracyclin	9.21	7.46	6.65	5.50
**n holdings**	**9,488**	**16,786**	**20,169**	**19,128**
**sum treatment frequencies in %**	**100**	**100**	**100**	**100**
**sum treatment frequencies**	**73,238.67**	**118,014.55**	**126,409.95**	**85,901.04**

* Cephalosporines of the 1st and 2nd generation as well as Paromomycin, phenoxymethylpen, difloxacin and sulfadoxin were not used in fattening pigs in this study.

We found that penicillines, especially amoxicillin, were an antibiotic class often used in all of the considered age groups in pigs. In fattening pigs, the treatment frequency of tetracyclines had a higher percentage per total treatments, but this drug class became less important with decreasing pig age. Polypeptides also had high shares of the total treatments in all age groups, with an obviously decreasing percentage, especially in sucklers and fattening pigs. Cephalosporins, especially ceftiofur, were relatively often used in sucklers, with an increasing percentage. Aminoglycosides and enrofloxacin (fluoroquinolones) had relatively high shares per overall treatment in sucklers, whereas lincomycin only played a role in fattening pigs. Potentiated sulfonamides had a small share of the total treatments in all age groups, and over the course of the half-years, the percentage decreased.

## Discussion

In the present investigation, we analysed the entire data set of the QS antibiotic monitoring system for pigs for the time period 1 July, 2013 to 31 June, 2015. The treatment frequencies were calculated and the percentages of active substances used per age group and time period were described.

There are two types of antibiotic consumption studies. On the one hand, there are full (nation-wide) surveys, such as Bos et al. [24]; DANMAP [4] or MARAN [6] and on the other hand, there are cross-sectional of longitudinal studies analysing a sample population of farms. These studies again may be separated into studies with a national for example Callens et al. [25], Merle et al. [12], Moreno [26] and van Rennings et al. [13] or a regional for example Timmerman et al. [20] or Sjölund et al. [27] perspective. While cross-sectional studies are always prone to a selection bias, this is usually not the case for full surveys. Our data is a full survey of all pig farms in Germany, which are members of the QS system. A farmer who wants to be part of the QS system has to fulfil different requirements [28]. Therefore, a membership bias cannot be ruled out completely, but it may be neglected in terms of the pork-production chain because this investigation has an excellent coverage of the pig population in Germany.

The evaluation period was two years and was aligned to half-year analyses due to the mandatory documentation duties derived from the German Pharmaceuticals Act. Others studies, such as Bos et al. [24], Sjölund et al. [23] and Trauffler et al. [29], looked at annual data, while for example van Rennings et al. [13] evaluated a time period of 100 days, which has to be taken into account when comparing the data.

In general, the underlying information of antibiotic consumption differs between the studies, which originate in different calculation and reporting methods. In the QS system, the data of the mandatory ADFs are recorded and used to calculate nUDD per age group.

For calculation, the number of animals treated and days treated is used directly from the forms (see Material and methods). This is in contrast to other systems, which have to estimate the animal weights by standard averages and/or the number of animals treated by assuming ADDs [e.g. 20, 25, 29], which both increase the uncertainty of the calculations.

An additional benefit of our investigation is that the use of antibiotics can be associated with an age group treated and the calculation method is not affected by varying dosages of antibiotics.

96.45% of the ADFs contained complete information regarding the variables needed to calculate the treatment frequency, a percentage stated as high quality for routine data. If one looked at robust statistical measures, like quartiles and alpha trimming for the percentile drug class, the data quality is sufficient for the analyses and interpretation suggested.

In addition to the aforementioned differences, there are more aspects that need to be kept in mind when comparing different studies, as well as participating farms. The stratification rules concerning production system and farm type are important, but differ among the different studies. In the present study, sucklers, weaners and fattening pigs are monitored separately; sows and boars are not taken into consideration. In different surveys, the various animal species and age groups were investigated in different combinations. For example, Sjölund et al. [23], and Trauffler et al. [29] analysed sucklers separately from sows. In some studies, sucklers and sows were analysed together [24], while in others, age groups were not differentiated [30]. Moreover, there are multi-species studies in which all livestock animals were analysed together [e.g. 7, 8]. We consider the approach in the present study to be useful. The different housing conditions and diseases of the three age groups resulted in a differing use of active substances and quantities. Therefore, a consideration with the present stratification seems to be more meaningful.

Also, the farm types of the participating farms are important. The consumption of antibiotics differs between specialised and non-specialised farms [31, 32]. In this study, all farm types were evaluated together, but in some surveys only farrow-to-finish farms were taken into account [23, 26].

In spite of all these differences, it is interesting to notice that the distribution of the different antibiotic active substances and drug classes per treatment is similar in various studies. In this study, beta lactams, tetracyclines and polypeptides show high shares of the total treatments in all age groups.

Sjölund et al. [23] showed that penicillines (benylpenicillin and amoxicillin) have a high share of the total treatments in all age groups, which corresponds to what we found for amoxicillin in our study. However, Sjölund et al. [23] included only the three most used active substances from every age group in their analyses, and the treatment is divided into individual and group treatments. Furthermore, in our study, a medial percentage of the treatment frequency of 3^rd^ and 4^th^ cephalosporins (about 12%) with an increasing trend was found in sucklers. In Sjölund et al. [23] no usage of cephalosporins in any of the considered age groups and participating holdings was documented because these antibiotics are not authorized in Sweden.

In a Belgian study, the UDD treatment incidence per 1,000 pigs at risk per day for different active substances was calculated and divided into oral and injectable. Proportionally, amoxicillin (30.0%) and colistin (30.7%) had the highest share in oral treatments. We found similar shares in treatment with these two active substances. Tulathromycin, macrolide, and Ceftifur LA, a cephalosporin, had the highest percentages of injectable treatments [25]. We saw a similar distribution in our data in sucklers, which were treated mainly with injectable preparations. In the present study the evaluation is done per active substance and although we include the long acting definition in our calculation we do not differentiate between long acting and other drugs in the results. However, a comparison between the values is not possible in detail, because Callens et al. [25] define fattening pigs as pigs between birth and slaughter and we divide the fattening period into three age groups.

Merle et al. [33] evaluated the antibiotic consumption data from Lower Saxony and North Rhine-Westphalia in Germany over a one-year period (from September 2006 until August 2007) from a sample of farms. Similar to our study, beta-lactams made up a great share of the total treatments in sucklers and fattening pigs, as well as the tetracyclines, which were used more often than beta-lactams in fattening pigs. In contrast to the present survey, Merle et al. [33] found that sulfonamides showed a relatively high percentage of the total treatments. One possible explanation is that veterinarians avoid potentiated sulfonamides because since 2014, potentiated sulfonamides are counted in Germany as two active substances in the official national antibiotic monitoring system. Trauffler et al. [29] focused on the use of the highest priority critically important antimicrobials defined by the WHO [34], and calculated the percentage of the total treatments of nADD_kg_/kg/year. They found that tylosin was the most used macrolide with 6.4% of the total treatments; percentages in the present study are similar to these findings. In total, the active substances classified as "highest priority critically important antimicrobials" have a small share of the total treatments in Trauffler et al. [29], as in our study, especially in the fluoroquinolones and in the cephalosporins in weaner and fattening pigs. The share of the macrolides in weaner and fattening pigs is higher as well as the share of the "highest priority critically important antimicrobials" in sucklers.

Apart from the "highest priority critically important antimicrobials," Trauffler et al. [29] also considered the other drug classes. We found similar percentages in both amoxicillin and colistin. Just as in this study, the share of potentiated sulfonamides found by Trauffler et al. [29] is small.

In summary, it is remarkable that penicillines and tetracyclines show a high share of the total treatments in several studies, despite various study approaches and calculation methods. Other drug classes were only used in small shares, such as cephalosporins, fluoroquinolones or pleuromutilins.

The descriptive analysis shows a decreasing trend in the quartiles of the treatment frequencies in fattening pigs. This trend may result from the rising public interest and the change in legislation in 2014, which was followed by a rethinking by veterinarians and farmers. The treatment strategy may have changed to more single treatments or to more vaccinations. It is also possible that animal health has improved through better animal hygiene and animal welfare, leading to a reduced need for antibiotic treatment. The recent launch of this surveillance system for sucklers and weaners in 2014 could be the reason a clear trend in these age groups has yet to be seen.

As the monitoring system for sucklers and weaners is in its initial phase, the data should be interpreted with a certain caution and no detailed statistical inference should be made at this point. The trends indicate a certain stability of the data, but its sustainability is uncertain. A continuation of the system is needed to get more reliable values, especially in sucklers and weaners. Further investigation can be done in the upcoming years.

In this study, holdings with no treatment in one or more half-years are found in all three age groups. Approximately 15% of the fattening pig holdings, and approximately 10% and 5% of the weaner and suckler holdings, respectively, do not get any antibiotics. Sjölund et al. [23] reported that all sucklers in their study were treated with antibiotics, but there were holdings of weaners (8%) and fattening pigs (5%) that were not treated. Moreno [26] observed around 6% of the sucklers, 2% of the weaners and 0% of the fattening pigs with no antibiotic treatment. Obviously, it is possible to raise pigs without or with few antibiotic treatments in the three considered age groups, although it is only a small percentage of holdings per half-year that manages without antibiotics.

## Conclusions

The calculation of the treatment frequency and of the percentage per active substance are appropriate methods to look at consumption and drug profile changes over time, but comparability with international studies is restricted. A reduction trend in total antibiotic usage can be seen in fattening pigs. In weaners and sucklers, clear trends cannot be observed to date, since the surveillance system, especially in sucklers and weaners, is still in the initial phase.
